# Acetylcarnitine Is Associated With Cardiovascular Disease Risk in Type 2 Diabetes Mellitus

**DOI:** 10.3389/fendo.2021.806819

**Published:** 2021-12-14

**Authors:** Shuo Zhao, Ming-Li Liu, Bing Huang, Fu-Rong Zhao, Ying Li, Xue-Ting Cui, Rong Lin

**Affiliations:** ^1^ Department of Pharmacology, School of Basic Medical Sciences, Xi’an Jiaotong University Health Science Center, Xi’an, China; ^2^ Human Resources Department, The First Affiliated Hospital of Jinzhou Medical University, Jinzhou, China; ^3^ Department of Sceintific Research, Dalian Runsheng Kangtai Medical Lab Co. Ltd., Dalian, China

**Keywords:** type 2 diabetes mellitus, cardiovascular disease, acylcarnitine, metabolism, relationship

## Abstract

**Objective:**

This study aimed to identify the association between specific short-chain acylcarnitines and cardiovascular disease (CVD) in type 2 diabetes mellitus (T2DM).

**Method:**

We retrieved 1,032 consecutive patients with T2DM who meet the inclusion and exclusion criteria from the same tertiary care center and extracted clinical information from electronic medical records from May 2015 to August 2016. A total of 356 T2DM patients with CVD and 676 T2DM patients without CVD were recruited. Venous blood samples were collected by finger puncture after 8 h fasting and stored as dried blood spots. Restricted cubic spline (RCS) analysis nested in binary logistic regression was used to identify possible cutoff points and obtain the odds ratios (ORs) and 95% confidence intervals (CIs) of short-chain acylcarnitines for CVD risk in T2DM. The Ryan–Holm step-down Bonferroni procedure was performed to adjust *p*-values. Stepwise forward selection was performed to estimate the effects of acylcarnitines on CVD risk.

**Result:**

The levels of C2, C4, and C6 were elevated and C5-OH was decreased in T2DM patients with CVD. Notably, only elevated C2 was still associated with increased CVD inT2DM after adjusting for potential confounders in the multivariable model (OR = 1.558, 95%CI = 1.124–2.159, *p* = 0.008). Furthermore, the association was independent of previous adjusted demographic and clinical factors after stepwise forward selection (OR = 1.562, 95%CI = 1.132–2.154, *p* = 0.007).

**Conclusions:**

Elevated C2 was associated with increased CVD risk in T2DM.

## Introduction

Type 2 diabetes mellitus (T2DM) is a major risk factor for cardiovascular disease (CVD) alongside other risk factors such as smoking and lipid disorders; in turn, CVD is the most prevalent cause of mortality among people with T2DM (1, 2). Individuals with diabetes have two to three times higher risk of developing CVD and two to four times increased risk of dying from heart diseases compared to their counterparts without diabetes (3–5).

Generally, obesity is a common risk factor for diabetes and its cardiovascular complications and has adverse effects on blood pressure and blood lipid (6). However, the body mass index (BMI) does not fully explain the increased risk of CVD in diabetics. Compared with the value of BMI, the metabolism and distribution characteristics of lipids will also have different effects on diabetes and CVD. For example, unlike fat that accumulates in the viscera, subcutaneous fat is negatively correlated with triglycerides (TGs), blood pressure, and atherosclerosis and has a protective effect on atherosclerosis (7). Besides, there is also a large variability of CVD risk for given fat mass. A review indicated that some lean people and obese people have similar risk of cardiovascular complications due to differences in fat distribution (8). Therefore, the risk of CVD in T2DM with diabetes cannot simply be measured by BMI. Interestingly, the heart energy of diabetic patients is mainly supplied by the oxidation of fatty acids (FAs) (9). As a small metabolic molecule, acylcarnitine is a product of FAs involved in heart energy supply (10). Acylcarnitine is essential for the transport of FAs into the mitochondria, which are free carnitines combined with coenzyme A (CoA) produced by FA β-oxidation (11–13). Meanwhile, acylcarnitine is also involved in the metabolism of triglycerides, cholesterol, and other lipids, as well as in the process of gluconeogenesis, which can comprehensively reflect the changes of glucose and lipid metabolism in diabetic patients.

A previous study found that the elevation of blood contents of short- and medium-chain acylcarnitines was related to the risk of CVD in T2DM (14). Animal experiments have also shown that the plasma levels of acetylcarnitine and hydroxybutyrylcarnitine in T2DM rats were increased. Further correlation analysis indicated that T2DM-related dyslipidemia was associated with the increased level of acetylcarnitine, and hydroxybutyrylcarnitine showed higher positive correlations with homeostasis model assessment of insulin resistance (HOMA-IR) (*R* > 0.64) (15). Similarly, a Russian study found that the levels of short-chain acylcarnitines, such as acetylcarnitine and isovalerylcarnitine, were higher in patients with CVD than those in non-CVD patients (16). These studies were consistent with our previous study to a certain extent, that some short-chain acylcarnitines might play an essential role in diabetic patients developing CVD. But only a few studies have investigated the association of these short-chain acylcarnitines and the risk of T2DM patients developing CVD.

Therefore, we carried out a cross-sectional study based on hospital patients with T2DM to explore the effects of short-chain acylcarnitines on CVD in T2DM patients.

## Methods and Results

### Research Design and Population

Electronic medical records were compiled to collect the demographic and disease information of patients in May 2015. By August 2016, we recruited 2554 T2DM patients from the First Affiliated Hospital of Liaoning Medical University. T2DM was diagnosed according to the 1999 criteria of the World Health Organization (17). We excluded the following patients: 1) aged <18 years, 2) with missing demographic information, 3) had disorders of consciousness, and 4) short-chain acylcarnitine metabolites were not determined, including C2, C3, C4, C4-OH, C4DC, C5, C5-OH, C5DC, C5:1, and C6. According to the standard, a total of 1,032 patients were enrolled in this study. The Ethics Committee for Clinical Research of the First Affiliated Hospital of Liaoning Medical University granted ethical approval for the study. Informed consent was waived due to the retrospective nature of the study, which is consistent with the Declaration of Helsinki.

### Data Collection and Definitions

Demographic information including age, gender, BMI, and duration of diabetes and drug use data such as of antidiabetic drugs, antihypertensive drugs, and lipid-lowering drugs were retrieved from electronic medical records. Trained doctors examined clinical data on systolic blood pressure (SBP), diastolic blood pressure (DBP), glycated hemoglobin (HbA1c), TGs, low-density lipoprotein cholesterol (LDL-C), and high-density lipoprotein cholesterol (HDL-C) in the laboratory of the hospital.

BMI was calculated as the weight at the last hospital visit (in kilograms) divided by the squared body height (in meters). Based on a previous research (18), BMI was divided into the following groups: underweight, <18.5 kg/m^2^; normal weight, 18.5–24 kg/m^2^; overweight, 24–28 kg/m^2^; obese, ≥28.0 kg/m^2^. Blood pressure was the average of two measurements using standard mercury sphygmomanometers after at least a 10-min rest in a sitting position. HbA1c ≥ 7% was defined as hyperglycemia. Dyslipidemia was diagnosed if TG ≥ 1.7 mmol/L, LDL-C ≥ 2.6 mmol/L, or HDL-C ≤ 1 mmol/L in males and HDL-C ≤ 1.3 mmol/L in females.

CVD was defined as having a medical history of coronary artery disease (CAD), heart failure, or stroke. Short-chain acylcarnitines include acetylcarnitine (C2), propionylcarnitine (C3), butyrylcarnitine (C4), hydroxylbutyrylcarnitine (C4-OH), succinylcarnitine (C4DC), isovalerylcarnitine (C5), 3-hydroxyisovalerylcarnitine (C5-OH), glutarylcarnitine (C5DC), tiglylcarnitine (C5:1), and hexanoylcarnitine (C6).

### Acylcarnitine Assays

Venous blood samples were collected by finger puncture after 8 h fasting and stored as dried blood spots. Experimental determination was taken at room temperature. Acylcarnitine was extracted from blood samples using the Millipore MultiScreen HV 96-well plate (Millipore, Billerica, MA, USA). Filtrate after extraction and quality control solutions were dried by pure nitrogen gas at 50°CC. Acylcarnitine quality control standards were purchased from Chromsystems (Grafelfing, Germany). Dried samples were dissolved by acetonitrile aqueous solution for final acylcarnitine assays. The assays were performed using an AB Sciex 4000 QTrap system (AB Sciex, Framingham, MA, USA). Acylcarnitine was scanned under positive mode. Analyst v1.6.0 software was applied for the system control and acylcarnitine data collection. Further data preprocessing is carried out through ChemoView 2.0.2 (AB Sciex).

### Statistical Analysis

All statistical results were conducted using Statistical Analysis System 9.4 version (SAS Institute Inc., Cary, NC, USA). Normality of continuous variables was tested by checking the quantile–quantile (Q–Q) plot. Continuous variables with normal distribution were expressed as the mean ± standard deviation (SD) and compared using Student’s *t*-test. Continuous variables with skewed distribution were expressed as medians (interquartile range) and compared using the Wilcoxon rank-sum test. Categorical variables were presented as quantity (percentage), and their differences between the CVD and non-CVD groups were compared using the chi-squared test or Fisher’s exact test. Binary logistic regression was performed to assess the odds ratios (ORs) and 95% confidence intervals (CIs) of the levels of acylcarnitine for the risk of CVD in T2DM. Restricted cubic spline (RCS) analysis embedded in logistic regression was used to examine the full-range associations between acylcarnitine levels and the risk of CVD in T2DM due to possible non-linearity. As usual, three knots were adopted in the study according to a previous study and the suggestion by Harrell (19–21). The ORs of acylcarnitines will change rapidly at some cutoff points if the relationship between acylcarnitines and CVD in T2DM is nonlinear. These cutoff points were used to stratify acylcarnitines into categorical variables by carefully observing the shape of the OR curves of acylcarnitine for CVD in T2DM. A two-step adjustment scheme was performed to control potential confounders. Firstly, we preliminarily created a univariable logistic model to explore the relation between acylcarnitines and CVD in T2DM. Secondly, we further adjusted the aforementioned factors consisting of age, gender, BMI, duration of diabetes, antidiabetic drugs, antidiabetic drugs, and lipid-lowering drugs, SBP, DBP, HbA1c, TG, LDL-C, and HDL-C. In addition, a stepwise forward selection was performed to identify acylcarnitines that had effects on CVD risk in TDM independent of these factors. Mean imputation and multiple imputation were performed to control for the impact of missing data in the multivariable model. Finally, *p*-values less than 0.05 were considered as statistically significant for all these analyses. The Bonferroni test was applied to adjust *p*-values for multiple comparisons.

## Results

### Demographic and Clinic Characteristics of Participants

Among all the study subjects, there were 356 T2DM patients suffering from CVD. Strokes were the most common (only diagnosed strokes: 193, 39.04%), followed by CAD (only diagnosed CAD: 111, 31.18%), and the least number of people suffered from heart failure (HF) (only diagnosed HF: 6, 1.69%). In both groups, there were more male than female patients. The age, duration of diabetes, SBP, and the proportions of intake of hypoglycemic drugs and lipid-lowering drugs in the CVD group were all greater than those in the non-CVD group. It was worth noting that, compared with the non-CVD group, the CVD group had more frequent HbA1c deletions, while the missing values of HDL-C, LDL-C, and TG were fewer. However, there were no statistically significant differences in the gender distribution, BMI, diastolic blood pressure, and intake of antidiabetic drugs between the two groups (**Table 1**).

**Table 1:** Demographic and clinical characteristics of diabetes according to occurrence of CVD.

	CVD (*n* = 356)	Non-CVD (*n* = 676)	*p*-value
Age (years)	65.00 (57.00–72.00)	54.00 (45.00–62.50)	<0.0001*
Gender, male	192 (53.93%)	357 (52.81%)	0.7313**
Duration of diabetes (years)	7.75 (2.00–12.00)	3.00 (0.00–10.00)	<0.0001*
BMI (kg/m^2^)	24.87 (22.87–7.34)	25.24 (22.65–7.68)	0.3671*
BMI categories			0.1441**
<18.5 (underweight)	10 (2.81%)	17 (2.51)	
18.5–24 (normal weight)	119 (33.43%)	235 (34.76%)	
24–28 (overweight)	163 (45.79%)	267 (39.50%)	
≥28 (obese)	64 (17.98%)	157 (23.22%)	
SBP (mmHg)	143.00 (130.00–161.00)	135.00 (122.00–150.00)	<0.0001*
DBP (mmHg)	80.00 (74.00–92.00)	82.00 (74.00–90.00)	0.5446*
HbA1c (%)	8.40 (7.50–10.60)	9.75 (7.90–11.10)	<0.0001*
HbA1c ≥ 7	180 (50.56%)	374 (55.33%)	0.0227**
HbA1c < 7	37 (10.39%)	40 (5.92%)	
Missing value	139 (39.05%)	262 (38.76%)	
HDL-C (mmol/L)	1.03 (0.85–1.26)	1.00 (0.84–1.24)	0.5748*
<1 in males or <1.3 in females	189 (53.09%)	305 (45.12%)	<0.0001**
≥1 in males or ≥1.3 in females	99 (27.81%)	148 (21.89%)	
Missing value	68 (19.10%)	223 (32.99%)	
LDL-C (mmol/L)	2.69 (2.09–3.26)	2.78 (2.25–3.44)	0.0097*
LDL-C ≥ 2.6	156 (43.54%)	279 (41.27%)	<0.0001**
LDL-C < 2.6	133 (37.36%)	174 (25.74%)	
Missing value	68 (19.10%)	223 (32.99%)	
TG (mmol/L)	1.62 (1.09–2.22)	1.69 (1.12–2.55)	0.0391*
TG ≥ 1.7	132 (37.08%)	229 (33.88%)	<0.0001**
TG < 1.7	156 (43.82%)	227 (33.58%)	
Missing value	68 (19.10%)	220 (32.54%)	
Antidiabetic drugs	292 (82.02%)	575 (85.06%)	0.2058**
Antihypertensive drugs	214 (60.11%)	199 (29.44%)	<0.0001**
Lipid-lowering drugs	189 (53.09%)	199 (29.44%)	<0.0001**
Only CAD	111 (31.18%)		
Only HF	6 (1.69%)		
Only stroke	139 (39.04%)		
CAD and stroke	59 (16.57%)		
CAD and HF	59 (16.57%)		
HF and stroke	20 (5.62%)		
CAD and stroke and HF	19 (5.34%)		

Data are represented as n (%), means ± standard deviation, or median (interquartile range).

CVD, cardiovascular disease; BMI, body mass index; SBP, systolic blood pressure; DBP, diastolic blood pressure; HbA1c, glycated hemoglobin; TG, triglyceride; LDL-C, low-density lipoprotein cholesterol; HDL-C, high-density lipoprotein cholesterol; CAD, coronary artery disease; HF, heart failure.

*P-values for comparisons between groups derived using Wilcoxon rank-sum test. **P-values for comparisons between groups derived using chi-squared test.

### Levels of Short-Chain Acylcarnitines Between the CVD and Non-CVD Groups

The levels of C2, C4, and C6 in the CVD group were higher than those in T2DM patients without CVD, whereas the concentration of C5-OH was lower. No significant differences in the levels of C3, C4-OH, C4DC, C5, C5DC, and C5:1 were found in both groups (**Table 2**).

**Table 2:** Short-chain acylcarnitine levels betweenT2DM patients with CVD and T2DM patients without CVD.

	CVD	Non-CVD	*p*-value
C2 (μmol/L)	12.540 (9.118–16.275)	11.320 (8.670–14.902)	0.0035
C3 (μmol/L)	1.355 (0.943–1.827)	1.373 (0.961–1.977)	0.2272
C4 (μmol/L)	0.212 (0.160–0.290)	0.199 (0.150–0.267)	0.0035
C4-OH (μmol/L)	0.101 (0.080–0.152)	0.108 (0.078–0.158)	0.5514
C4-DC (μmol/L)	0.660 (0.518–0.860)	0.638 (0.470–0.840)	0.1236
C5 (μmol/L)	0.146 (0.110–0.199)	0.150 (0.110–0.195)	0.9172
C5-OH (μmol/L)	0.248 (0.188–0.353)	0.270 (0.208–0.350)	0.0081
C5DC (μmol/L)	0.083 (0.060–0.126)	0.080 (0.050–0.118)	0.1178
C5:1 (μmol/L)	0.060 (0.047–0.082)	0.060 (0.048–0.080)	0.4233
C6 (μmol/L)	0.057 (0.040–0.070)	0.0480 (0.033–0.063)	<0.0001

P-values for comparisons between groups were derived using the Wilcoxon rank-sum test.

CVD, cardiovascular disease; T2DM, type 2 diabetes mellitus; C2, acetylcarnitine; C3, propionylcarnitine; C4, butyrylcarnitine; C4-OH, hydroxylbutyrylcarnitine; C4DC, succinylcarnitine; C5, isovalerylcarnitine; C5-OH, 3-hydroxyisovalerylcarnitine; C5DC, glutarylcarnitine; C5:1, tiglylcarnitine; C6, hexanoylcarnitine.

### Associations of Short-Chain Acylcarnitine and Risk of CVD in T2DM

**Figure 1:**
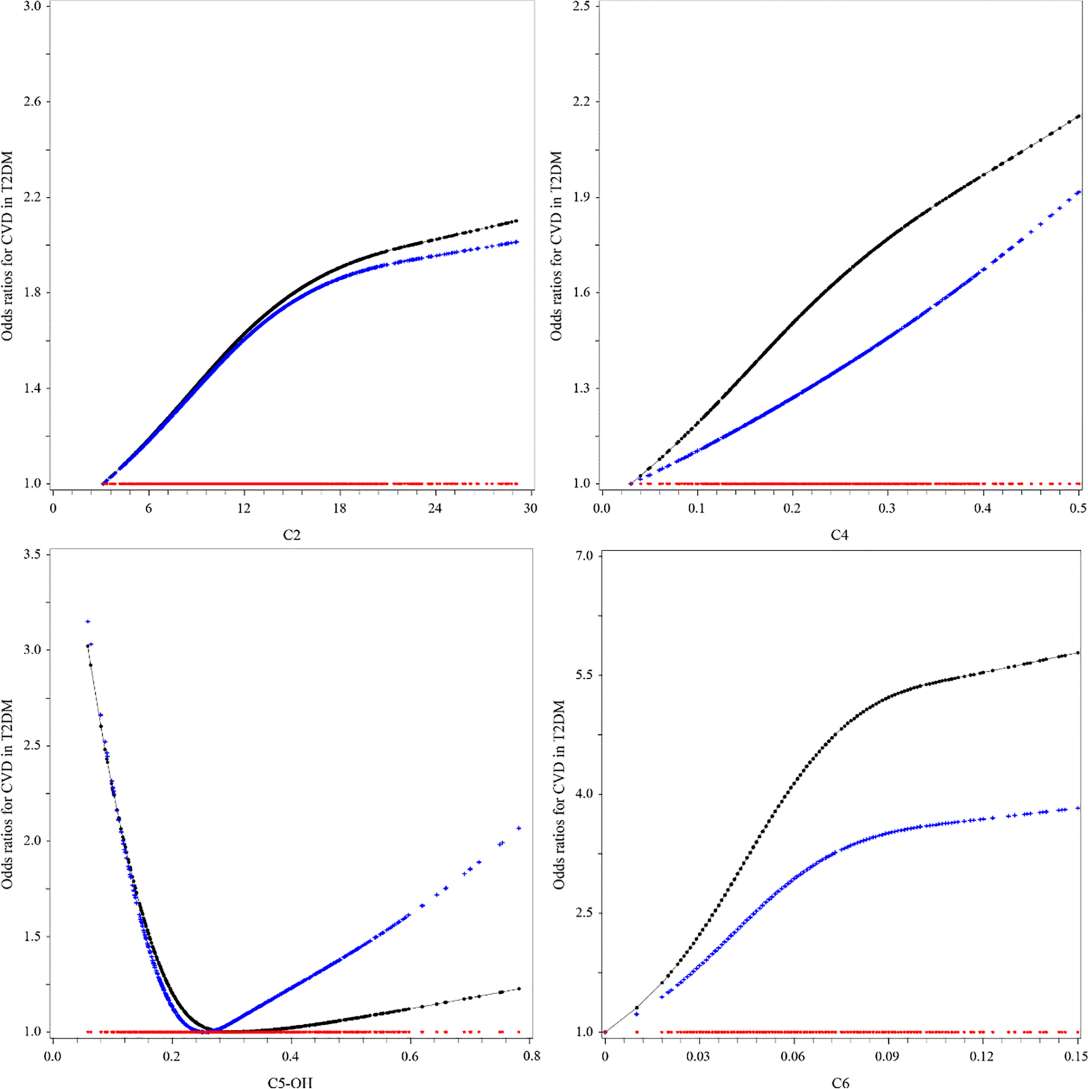
Associations between short-chain acylcarnitine and the risk of cardiovascular disease (CVD) in type 2 diabetes mellitus (T2DM). The black lines were derived from univariable model 1, the blue lines from multivariable analyses (see **Table 3**, multivariable model 2, for the list of adjusted variables), and the red lines were the reference lines at OR = 1.

Except for C4, the relationships between C2, C5-OH, and C6 and CVD in T2DM were nonlinear by observing the OR curves (**Figure 1**). C2 and C4 were positively associated with the risk of CVD in T2DM in the univariable model (model 1). The ORs (95%CI) were as follows: C2 = 1.576 (1.203–2.064) and C4 = 3.551 (1.409–8.946). The relationship between C5-OH and CVD was a U-shaped curve. In the univariable analysis, C5-OH less than 0.22 μmol/L was negatively associated with CVD in T2DM, utilizing 0.22–0.30 μmol/L as the reference [OR (95%CI) of C5-OH for <0.22 *vs*. 0. 22–0.30 μmol/L: 1.638 (1.181–2.270)]. Only the association of C2 with CVD in T2DM was still statistically significant after adjusting for other potential confounders in the multivariable model (model 2) (C2: OR = 1.558, 95%CI = 1.124–2.159, *p* = 0.0078, Bonferroni-adjusted *p* = 0.0125). The OR of C2 for CVD in T2DM only had a slight change, which was independent of traditional risks in the stepwise selection procedure (C2: OR = 1.562, 95%CI = 1.132–2.154) (**Table 3**). Moreover, this relationship also did not change statistically after filling in the missing values (**Supplementary Table S1**).

**Table 3:** Odds ratios of short-chain acylcarnitine for CVD risk in T2DM.

	OR	95%CI	*p*-value
Model 1			
C2 ≥ *vs*. <14 μmol/L	1.576	1.203–2.064	0.0009
C4 (μmol/L)	3.551	1.409–8.946	0.0072
C5-OH (μmol/L)			
<0.22	1.638	1.181–2.270	0.0031
≥0.22 to ≤0.30	Reference		
>0.30	1.023	0.738–1.419	0.8906
C6 ≥ *vs*. <0.08 μmol/L	0.893	0.404–1.970	0.7785
Model 2			
C2 ≥ *vs*. <14 μmol/L	1.558	1.124–2.159	0.0078
C4 (μmol/L)	3.727	1.220–11.388	0.0210
C5-OH (μmol/L)			
<0.22	1.614	1.091–2.387	0.0165
≥0.22 to ≤0.32	Reference		
>0.32	1.295	0.876–1.916	0.1952
C6 ≥ *vs*. <0.08 μmol/L	0.487	0.196–1.209	0.1208
Stepwise regression			
C2 ≥ *vs*. <14 μmol/L	1.562	1.132–2.154	0.0067

Model 1 is the univariable model. Model 2 is the multivariable model, further adjusted for age, sex, body mass index, duration of diabetes, glycated hemoglobin, systolic blood pressure, diastolic blood pressure, triglyceride, low-density lipoprotein cholesterol, high-density lipoprotein, antidiabetic drugs, lipid-lowering drugs, and antihypertensive drugs. Values in bold are p-values less than 0.0125 (Bonferroni = 0.05/4) for the association of acylcarnitine with CVD in T2DM.

CVD, cardiovascular disease; T2DM, type 2 diabetes mellitus; C2, acetylcarnitine; C4, butyrylcarnitine; C5-OH, 3-hydroxyisovalerylcarnitine; C6, hexanoylcarnitine.

## Discussion

Our study found that the levels of C2, C4, and C6 in short-chain acylcarnitine increased in T2DM patients with CVD, while the level of C5-OH was decreased. Additionally, an elevated C2 level was associated with an increased risk of CVD in T2DM after considering demographic and clinical factors.

C2 is a small metabolic molecule in the body that plays a vital role in cellular respiration. It removes acetyl groups from cells and participates in energy transfer. C2 serves as a transmission tool for FAs to enter the mitochondria for the production of ATP during cellular respiration (22).

Specifically, free FAs are activated in the cytoplasm to form acyl-CoA. Acyl-CoA enters the mitochondria by carnitine shuttle through carnitine palmitoyl transferase 1 (CPT1) and carnitine palmitoyltransferase2 (CPT2) on the mitochondrial membrane (23). After entering the mitochondrial matrix, acyl-CoA is dehydrogenated, added water, dehydrogenated again, and sulfated under the catalysis of the FA oxidase system (24). Finally, acyl-CoA is broken to form one molecule of acetyl-CoA and one molecule of two-carbon chain shortened acyl-CoA (25). The shortened acyl-CoA repeats the above process to generate acetyl-CoA or combine with free carnitine to form acyl-carnitine. Acetyl-CoA is further involved in the synthesis pathway of the following substances (26): 1) acetyl-CoA and free carnitine are converted into acetylcarnitine by carnitine acetyltransferase (CRAT); 2) acetyl-CoA participates in the tricarboxylic acid (TCA) cycle to provide ATP; 3) acetyl-CoA as a direct raw material to synthesize cholesterol; 4) acetyl-CoA is converted into a ketone body.

It was worth noting that a US study indicated that CPT-I α-subtype mRNA abundance was increased in the heart of diabetic or fasted rats (27). Meanwhile, increased FA uptake associated with reduced glucose oxidation has been observed in the heart of both diabetic mice (*Ob*/*ob* and *db*/*db* mice) and in patients with type 2 diabetes (28–30). The characteristic of a diabetic heart is that the supply of substrates exceeds the need for TCA cycle (31). A study found that the genes involved in glucose transport and utilization were inhibited and that significant heart failure occurred at 4 weeks of age in a lipid-toxic mouse (the FA uptake rate exceeded the utilization rate), which was constructed using transgenic technology (32). Furthermore, an increase in the acetyl-CoA/CoA ratio was discovered in the heart of patients with diabetic cardiomyopathy (33).

**Figure 2:**
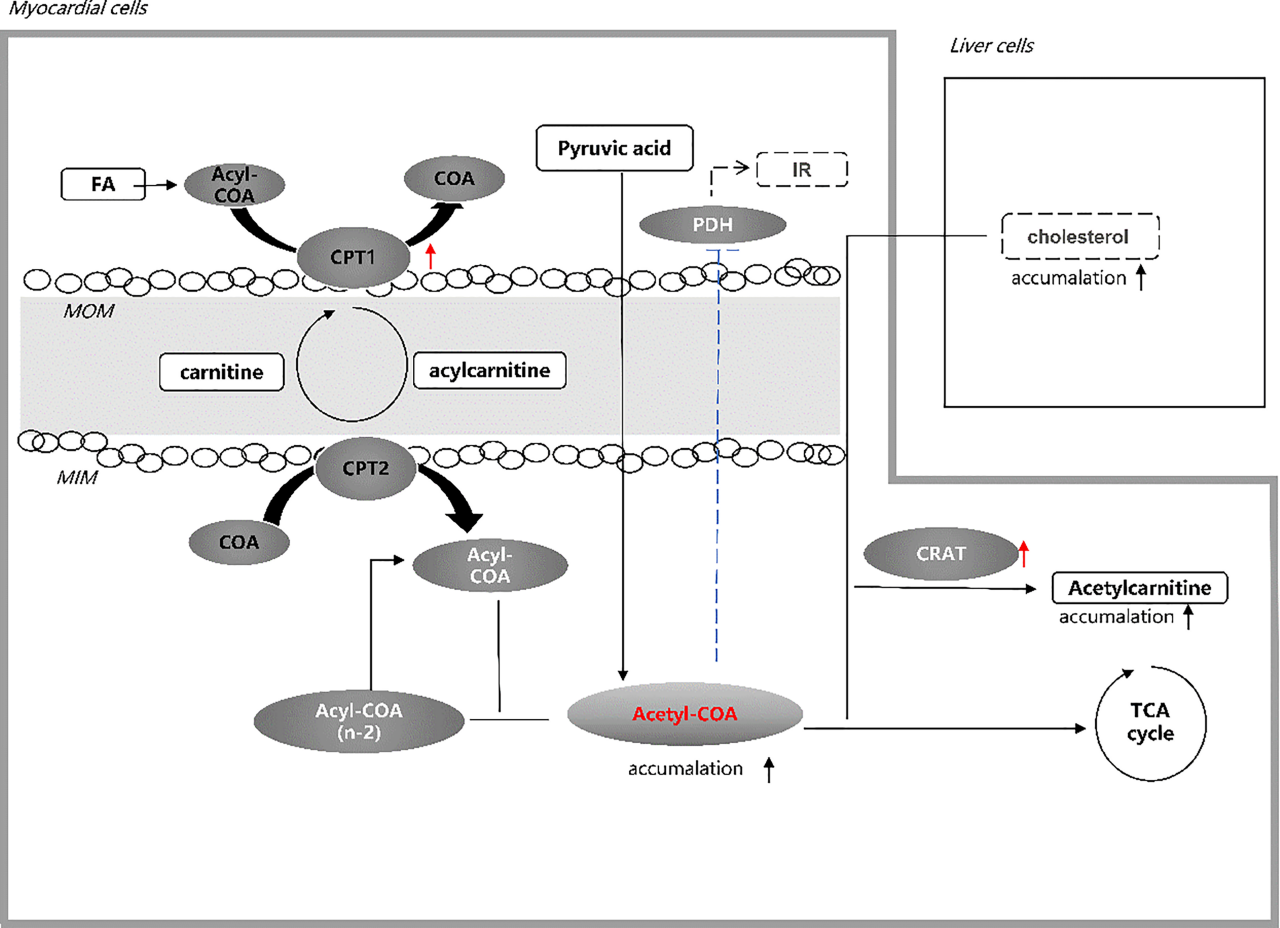
Hypothesis of acetylcarnitine association with CVD in T2DM. Elevated acetylcarnitine due to the higher activity of CPT1 and CRAT in patients with CVD in T2DM may reflect the state of accumulation of acetyl-CoA exceeding the capacity of the TCA cycle. Increased level of acetyl-CoA in the heart of T2DM patients leads to intensified insulin resistance by inhibiting PDH and increased cholesterol production in liver cells. Both pathways may account for the high CVD risk in T2DM. *FA*, fatty acid; *CoA*, co-enzyme A; *CPT 1*, carnitine palmitoyl transferase 1; *MOM*, mitochondrial outer membrane; *MIN*, mitochondrial inner membrane; *CPT 2*, carnitine palmitoyl transferase 2; *IR*, insulin resistance; *PDH*, pyruvate dehydrogenase; *CRAT*, carnitine acetyltransferase; *TCA*, tricarboxylic acid.

Therefore, we suspect that the accumulation of acetyl-CoA is caused by the FA uptake rate exceeding the need of the TCA cycle rate. Besides, a cohort study found that a higher transcriptional level of CRAT and elevated C2 were observed in impaired glucose tolerance (IGT) and T2DM patients, which presented during follow-up (34). The higher CRAT transcription level may explain the increase of acetylcarnitine due to a part of accumulation of acetyl-CoA (35). Moreover, the glycolysis pathway from pyruvate to acetyl-CoA will be further inhibited by negative feedback when excess acetyl-CoA exceeds the conversion capacity of the TCA cycle, thus exacerbating insulin resistance (36). Normal insulin signaling in the vascular endothelium can protect against atherosclerosis (37). Insulin resistance is associated with reduced production and release of nitric oxide in vascular endothelial cells (38), leading to impaired cellular function and, thus, an increased risk of atherosclerosis (39). In addition, as a direct precursor, the accumulation of acetyl-CoA may lead to increased cholesterol production in the liver, which may exceed the body’s metabolic capacity, deposit in the blood vessels, leading to atherosclerosis (**Figure 2**). A study involving 1,185 hospitalized patients with suspected coronary heart disease (CHD) found that elevated C2 worsened coronary stenosis after coronary angiography (OR = 1.778, 95%CI = 1.106–2.857) (40). Other population studies have demonstrated that C2 is associated with higher severity of CHD and adverse cardiovascular outcomes (41–44).

There were several shortcomings and limitations in our study. Firstly, it was difficult to determine the causal relationship between acylcarnitine and CVD in T2DM due to the cross-sectional nature of the study. Secondly, the levels of short-chain acylcarnitine are influenced by dietary habits. Since we retrospectively retrieved the data from the hospital’s electronic medical records, information regarding diet was not available for further investigation. In order to avoid the bias caused by diet as much as possible, we used fasting samples and adjusted the BMI, LDL-C, HDL-C, and TG. Thirdly, further enzyme analysis was needed to assay the levels of C2-related enzymes to support the conjecture involving related enzymes. Last but not least, the presence of missing values in our lipid index might affect the stability of the results. We reduced this effect by converting missing variables into categorical variables in the analysis. Also, the relationship between C2 and CVD in T2DM did not change significantly after mean and multiple imputations.

There are public health significance and clinical implications in our research. In combination with previous studies, we determined that C2, as a novel target for CVD in T2DM, may reflect the state of acetyl CoA accumulation in T2DM patients with CVD. C2 reflected acetyl-CoA, and its downstream metabolites are helpful in further exploring the pathogenesis of cardiovascular diseases.

In summary, an elevated level of C2 is associated with an increased risk of CVD in T2DM. Prospective studies are needed to further validate this finding and explore the pathological mechanism of CVD in T2DM.

## Data Availability Statement

The raw data supporting the conclusions of this article will be made available by the authors, without undue reservation.

## Ethics Statement

The studies involving human participants were reviewed and approved by the First Affiliated Hospital of Liaoning Medical University. The patients/participants provided written informed consent to participate in this study. Written informed consent was obtained from the individual(s) for the publication of any potentially identifiable images or data included in this article.

## Author Contributions

RL, M-LL, BH, and X-TC designed the study. SZ and M-LL analyzed the data and wrote the draft. RL, BH, F-RZ, YL, X-TC, and SZ gave critical comments and contributed to the writing of this manuscript. All authors contributed to the article and approved the submitted version.

## Funding

This research was financially supported by the Development and Application of Clinical Mass Spectrometry Detection Technology for Important Small Molecule Metabolites (2020JH2/10300116) and the Key R&D Program of Liaoning Province: platform name: Dalian Laboratory Medicine Mass Spectrometry Technology Innovation Center.

## Conflict of Interest

Authors BH, X-TC, M-LL, F-RZ, and YL were employed by the company Dalian Runsheng Kangtai Medical Lab Co. Ltd.

The remaining authors declare that the research was conducted in the absence of any commercial or financial relationships that could be construed as a potential conflict of interest.

## Publisher’s Note

All claims expressed in this article are solely those of the authors and do not necessarily represent those of their affiliated organizations, or those of the publisher, the editors and the reviewers. Any product that may be evaluated in this article, or claim that may be made by its manufacturer, is not guaranteed or endorsed by the publisher.

## Abbreviations

CVD (cardiovascular disease) was defined as having a history of coronary artery disease, heart failure or stroke. Short-chain acylcarnitines: acetylcarnitine (C2); propionylcarnitine (C3); butyrylcarnitine (C4); hydroxylbutyrylcarnitine (C4-OH); succinylcarnitine (C4DC); isovalerylcarnitine (C5); 3-hydroxyisovalerylcarnitine (C5-OH); glutarylcarnitine (C5DC); tiglylcarnitine (C5:1); hexanoylcarnitine (C6); Demographic information T2DM, type2 diabetes mellitus; BMI, body mass index; FA, fatty acid; TG, triglyceride; COA, coenzyme A; hexanoylcarnitine; SBP, systolic blood pressure; DBP, diastolic blood pressure, HbA1c, glycated hemoglobin; LDL-C, low density lipoprotein cholesterol; HDL-C, high density lipoprotein cholesterol; OR, odds ratio; CI, confidence interval; CAD, coronary artery disease; SD, standard deviation; CPT1, carnitine palmitoyl transferase 1; CPT2, carnitine palmitoyltransferase2; CRAT, carnitine acetyltransferase; CHD, coronary heart disease.
