# Supplementary material for: Acetylcarnitine Is Associated With Cardiovascular Disease Risk in Type 2 Diabetes Mellitus
Source: Front Endocrinol (Lausanne). 2021 Dec 14;12:806819. doi: 10.3389/fendo.2021.806819 (PMC8712495; doi:10.3389/fendo.2021.806819)
Supplement: Supplementary file 1 [file Table_1.docx]

Supplement table1:Odds ratios of short-chain acylcarnitineafter mean imputation and multiple imputation.

|  |  | Mean imputation | | | Multiple imputation | | |
| --- | --- | --- | --- | --- | --- | --- | --- |
|  |  | OR | 95%CI | P-Value | OR | 95%CI | P-Value |
| Model 2A | C2≥ VS. <14 μmol/L | 1.544 | 1.112 -1.143 | **0.0095** | 1.550 | 1.124 -2.138 | **0.0075** |
|  | C4, μmol/L | 3.817 | 1.246-11.692 | 0.0190 | 3.813 | 1.250-11.630 | 0.0187 |
|  | C5-OH, μmol/L |  |  |  |  |  |  |
|  | <0.22 | 1.572 | 1.061- 2.328 | 0.0240 | 1.530 | 1.039-2.251 | 0.0311 |
|  | ≥0. 22~≤0.30 | ref |  |  | ref |  |  |
|  | >0.30 | 1.290 | 0.872- 1.908 | 0.2025 | 1.270 | 0.862- 1.871 | 0.2262 |
|  | C6≥ VS.<0.08, μmol/L | 0.550 | 0.215- 1.407 | 0.2124 | 0.443 | 0.180- 1.086 | 0.0752 |
| Stepwise regression |  |  |  |  |  |  |  |
|  | C2≥ VS. <14, μmol/L | 1.552 | 1.124- 2.145 | 0.0077 | 1.553 | 1.132- 2.131 | 0.0064 |

Model 2A adjusted same factors of model 2 in table 2 after mean imputation and multiple imputation.

Boldface type indicates P value less than 0.0125 (Bonferroni: 0.05/4) for the association of acylcarnitine with CVD in T2DM.
